# A Selective Oxidation Strategy towards the Yolk–Shell Structured ZnS@C Material for Ultra-Stable Li-Ion Storage

**DOI:** 10.3390/ma16052097

**Published:** 2023-03-04

**Authors:** Wenhua Liao, Qianqian Hu, Xiaoshan Lin, Ruibo Yan, Guanghao Zhan, Xiaohui Wu, Xiaoying Huang

**Affiliations:** 1 State Key Laboratory of Structural Chemistry, Fujian Institute of Research on the Structure of Matter, The Chinese Academy of Sciences, Fuzhou 350002, China; 2 College of Chemistry and Materials Science, Fujian Normal University, Fuzhou 350007, China

**Keywords:** yolk-shell structure, ZnS@C composite, selective oxidation, electrochemical properties, lithium-ion battery

## Abstract

Metal chalcogenides are attractive anode materials for lithium-ion batteries (LIBs) due to their high theoretical capacities. With the advantages of low cost and abundance reserves, ZnS is regarded as the prime candidate anode material for future generations, but its practical application is hindered by the large volume expansion during repeated cycling processes and inherent poor conductivity. Rational design of the microstructure with large pore volume and high specific surface area is of great significance to solve these problems. Here, a carbon-coated ZnS yolk-shell structure (YS-ZnS@C) has been prepared by selective partial oxidation of a core-shell structured ZnS@C precursor in air and subsequent acid etching. Studies show that the carbon wrapping and proper etching to bring cavities can not only improve the material’s electrical conductivity, but can also effectively alleviate the volume expansion problem of ZnS during its cycles. As a LIB anode material, the YS-ZnS@C exhibits an obvious superiority in capacity and cycle life compared to ZnS@C. The YS-ZnS@C composite shows a discharge capacity of 910 mA h g^−1^ at the current density of 100 mA g^−1^ after 65 cycles, compared to only 604 mA h g^−1^ for ZnS@C after 65 cycles. Notably, at a large current density of 3000 mA g^−1^, a capacity of 206 mA h g^−1^ can still be maintained after 1000 cycles (over three times of the capacity for ZnS@C). It is expected that the synthetic strategy developed here is applicable to designing various high-performance metal chalcogenide-based anode materials for LIBs.

## 1. Introduction

LIBs have been widely used in various portable devices and electric vehicles [1,2,3]. This commercial graphitic material has been extensively used in LIBs as an anode material due to its stable cycling performance. However, its low theoretical specific capacity is inconsistent with the demand of light weight design, high energy density and power capacity of current LIBs [1,2]. Massive efforts have been invested to further boost novel anode materials of LIBs. To this end, metal chalcogenides with higher theoretical capacity are one of the research hotspots. Metal chalcogenides like FeS [4,5], MoS_2_ [6,7], Ni_3_S_4_ [8], VS_2_ [9], In_2_S_3_ [10], CoS_2_ [11,12], SnS [13,14,15], Bi_2_S_3_ [16], MnS [17,18], Sb_2_S_3_ [19,20], Cu*_x_*S [21], and ZnS [22,23] indeed exhibit significantly high stability and high capacity. Especially, ZnS acting as the anode material for LIBs has advantages of low cost and abundance reserves, which exhibit great potential in next generation anode materials [22,23]. However, some common challenges remain to be addressed for individual ZnS as the LIB electrode. The foremost is the large volume expansion during the cycling processes, which causes the fragmentation of microstructure and the formation of unstable solid electrolyte interphase (SEI) film, leading to poor capacity retention and poor cycle stability [22,23,24,25].

The core-shell structure formed by carbon-encapsulated metal sulfides can effectively improve the conductivity of the material and inhibit the loss of active substances. However, that still cannot alleviate its volume expansion problem. Yolk-shell structure is a branched subfield of core-shell structure that has attracted wide attention because of its large specific surface area, low density, easy functionalization of the inner core, and good molecular loading [26]. It has the unique morphological characteristics of a movable core in the hollow shell, which can effectively solve the problems of volume expansion and cycle decay of Li-ion battery electrodes by exploiting the advantages of the regulatable internal space [27]. Usually, the Yolk-shell structure is obtained by first coating the SiO_2_ layer and the carbon layer on the compound, and then removing the intermediate template layer SiO_2_ [28]. For instance, Guan et al. [29] prepared a Fe_3_O_4_@C yolk-shell structure composite material by this strategy, and a capacity of 680 mA h g^−1^ and stable cycle performance were obtained. Wan et al. [30] designed an yolk-shell structure composite with Sn wrapped in the hollow carbon spheres, which could effectively prevent electrode pulverization and adapt to the volume expansion; as a result, the capacity was greater than 550 mA h g^−1^ after 100 cycles. Similarly, the design of yolk-shell structure can also effectively alleviate volume expansion issues during charge-discharge processes in some metal chalcogenides, such as Co_9_S_8_ [31], CoS_2_ [32], FeS [33], Zn-Fe-S [34], FeS_2_ [35], (Fe_0.5_Ni_0.5_)_9_S_8_ [36], MoS_2_/Ni_3_S_2_ [37], and Sb_2_S_3_ [38], and thus can improve their electrochemical performances. Therefore, rationally designing the ZnS based anode material with Yolk-shell structure is expected to effectively solve its volume expansion and contraction issues, improving its service life and rate performance in LIBs. Xu et al. [39] and Wang et al. [23] have synthesized yolk-shell structures of ZnS/C materials from templates of Zn_2_GeO_4_ and ZIF-8, respectively, however, additional sulfidation treatments were required. Therefore, a simpler and more convenient synthetic method is to be developed.

Here, a carbon-coated ZnS yolk-shell structure (YS-ZnS@C) was prepared by selective partial oxidation of the core shell structure of ZnS@C in air to obtain ZnS/ZnO@C materials and further acid etching of the ZnS/ZnO@C to remove ZnO. Since the weakness of volume expansion and electrical conductivity were solved properly by the rational design of microstructure, the obtained YS-ZnS@C as the LIB anode material exhibits the high reversible capacity and excellent cyclic stability at the high current density. To our knowledge, the selective oxidation strategy towards yolk-shell structured ZnS@C composite as LIB anode material hasn’t been reported before.

## 2. Synthesis

Synthesis of ZnS@C material with a core shell structure. Core shell structure of ZnS@C material was prepared by our previously reported synthetic method [40], and the specific synthesis process has been described in detail in the supporting information.

Synthesis of YS-ZnS@C materials with a yolk-shell structure. The obtained ZnS@C (ca. 0.30 g) was oxidized in air at a certain temperature (e.g., 420 °C) for a certain number of hours (2–20 h) to convert the ZnS@C to ZnS/ZnO@C. The as-obtained ZnS/ZnO@C composites were corroded with 5 wt% acetic acid (HAc) solution for 0.5 h, which was washed with water and ethanol, and then collected by centrifugation and dried at 80 °C to obtain YS-ZnS@C materials. The ZnS/ZnO@C composites obtained by oxidation at 420 °C for 4 h and 8 h are named as ZnS/ZnO@C-1 and ZnS/ZnO@C-2, respectively; and the corresponding YS-ZnS@C materials are named as YS1-ZnS@C and YS2-ZnS@C, respectively.

The details of materials, characterizations, and electrochemical measurements can be found in the supporting information.

## 3. Results and Discussions

**Figure 1:**
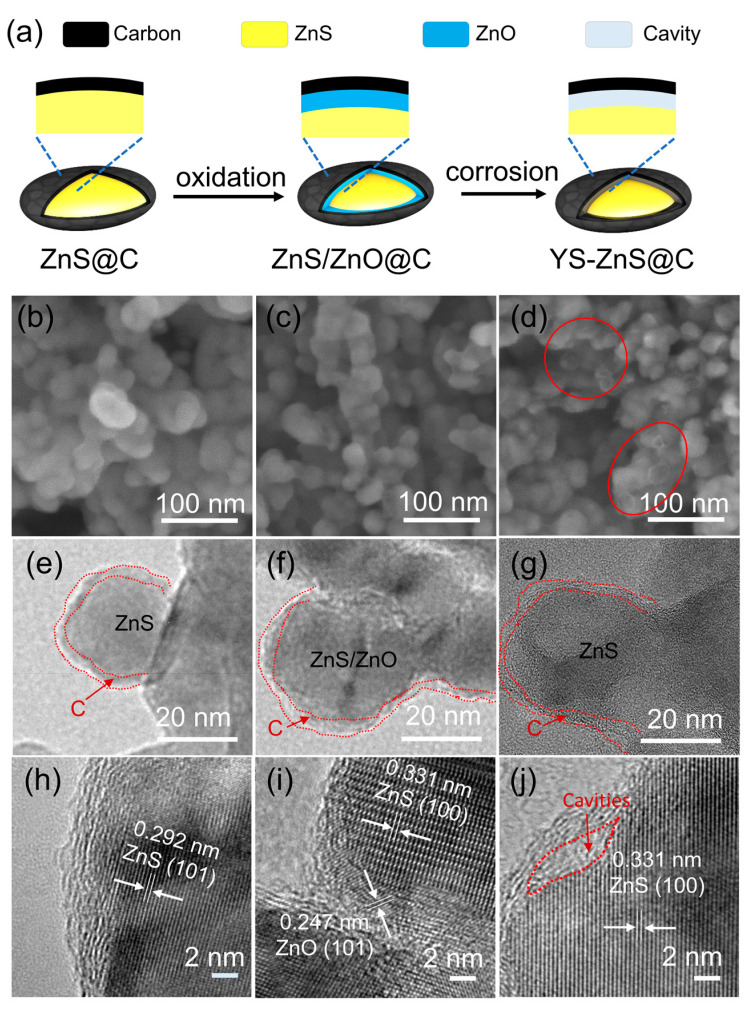
(**a**) Schematic illustrations for the formation process of yolk-shell structured ZnS@C. SEM images of (**b**) ZnS@C, (**c**) ZnS/ZnO@C-1, and (**d**) YS1-ZnS@C. TEM images of (**e**) ZnS@C, (**f**) ZnS/ZnO@C-1, and (**g**) YS1-ZnS@C. HRTEM images of (**h**) ZnS@C, (**i**) ZnS/ZnO@C-1, and (**j**) YS1-ZnS@C.

A facile fabrication procedure for a yolk-shell structure of ZnS@C via a selective oxidation strategy is illustrated in Figure 1a. A pre-prepared ZnS@C via vacuum pyrolysis method was used as a precursor, in which the ZnS was tightly wrapped with a uniform carbon layer. Calcining the ZnS@C at 420 °C in air results in the partial oxidation of ZnS to form ZnO/ZnS@C. Then, the acetic acid was used to corrode ZnO to fabricate the buffer space beyond the carbon layer, obtaining the YS-ZnS@C. The structure and morphology of as-prepared composites of ZnS@C, ZnS/ZnO@C-1, and YS1-ZnS@C were confirmed by the scanning electron microscopy (SEM) and transmission electron microscopy (TEM) images as shown in Figure 1b–j and Figure S1. They show that the size of the particles is ~30 nm, and the thickness of the carbon layer is ~3–5 nm. The ZnS and ZnO/ZnS are tightly coated by carbon layers in the ZnS@C and ZnS/ZnO@C-1, respectively, while an obvious buffer space between ZnS and C in the yolk-shell structure of YS1-ZnS@C was observed. Furthermore, as the interstices exist between the carbon layer and ZnS, it was observed in SEM image that the particle surface seemed to be wrapped with a layer of ‘white silk’ (marked with red circles in Figure 1d), which is in agreement with previous reports [41]. The lattice spacing of 0.292 and 0.331 nm are attributed to the (101) and (100) planes of Würtzite ZnS, respectively, as shown in Figure 1h–j. The lattice spacing of 0.247 nm corresponding to (101) plane of hexagonal ZnO can be found in the ZnS/ZnO@C-1 (Figure 1i), which finally disappears in the YS1-ZnS@C due to the acid etching (Figure 1j).

**Figure 2:**
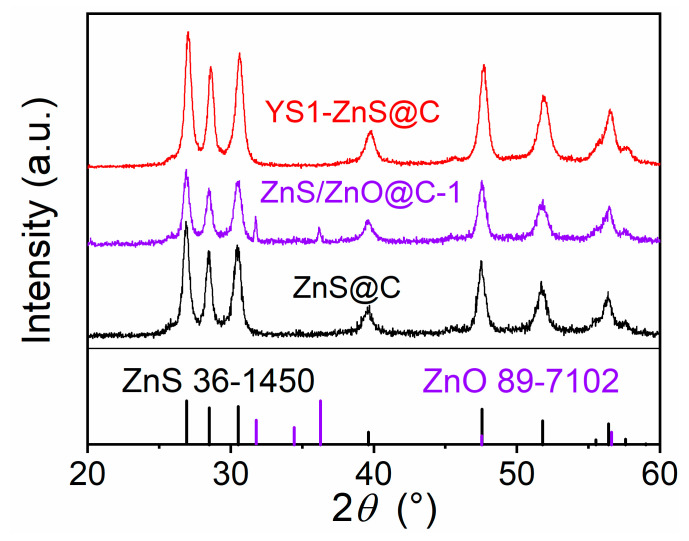
XRD patterns of ZnS@C, ZnS/ZnO@C-1 and YS1-ZnS@C.

To explore the structure evolution of these composites, X-ray diffraction (XRD) characterization was further adopted. Figure 2 and Figure S2a show the XRD patterns of ZnS@C, ZnS/ZnO@C-1, ZnS/ZnO@C-2, YS1-ZnS@C, and YS2-ZnS@C. The pattern of ZnS@C shows that the peaks located at 2*θ* = 26.9°, 28.5°, 30.5°, 39.6°, 47.6°, and 51.8° can be assigned to (100), (002), (101), (102), (110), and (103) crystallographic planes of the Würtzite ZnS phase (PDF.36-1450), respectively. After partial oxidation, additional mini diffraction peaks located at 2*θ* = 31.8°, 34.4°, 36.3°, 47.5°, and 56.6° were found in ZnS/ZnO@C-1 and ZnS/ZnO@C-2, corresponding to the (100), (002), (101), (102), and (110) crystallographic planes of hexagonal (*P*6_3_*mc*) ZnO (PDF. 89-7120), respectively. No characteristic peaks of ZnO are detected in the YS1-ZnS@C or YS2-ZnS@C, indicating that the ZnO was completely etched away. Besides, we find that the heat treatment duration also plays a particularly important role in the structural transformation of ZnS@C. Obviously, as shown in Figure S2b, no diffraction peaks of ZnO are found at an oxidation temperature of 410 °C, while the apparent peaks of ZnO can be found under the oxidation temperature of 420 °C. With the rising of calcination temperature, the intensities of diffraction peaks of ZnO increases gradually. As the temperature was raised to 500 °C, the XRD intensity of ZnO is obviously higher than that of ZnS. Discernibly, the color of the obtained ZnS/ZnO@C changes gradually from dark black to greyish white with the increasing of calcination temperature.

**Figure 3:**
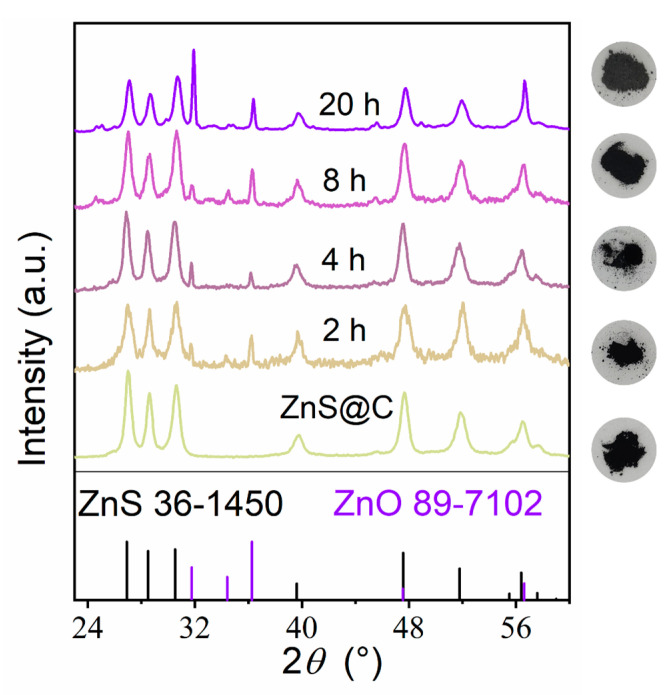
XRD patterns and photos of the ZnS@C samples after oxidation at 420 °C for various time.

Moreover, the effect of oxidation duration on the structural transformation of ZnS/ZnO@C has also been explored. Figure 3 shows the XRD patterns of the ZnS@C after oxidation at 420 °C for various time. The diffraction peaks of ZnS and ZnO for ZnS@C after oxidation at 420 °C for 2–8 h are clearly distinguished, and the diffraction intensity of ZnS is obviously higher than that of ZnO. However, after oxidation for 20 h, the diffraction intensity of ZnO is distinctly higher than that of ZnS. The phenomenon of enhanced ZnO peak intensity may be ascribed to the increase of oxidation time which leads to increased crystallinity or improved ZnO content in ZnS/ZnO@C. Interestingly, its components have also undergone subtle changes. Elemental analysis (EA) results show that the O content of ZnS/ZnO@C increases while the C content of samples decreases with the increase of oxidation duration (Table S1). These results inspire us to further investigate the thermodynamics of composites. The main reactions of the composite are the oxidations of zinc sulfide and carbon that produce zinc oxide and carbon dioxide, respectively, as follows:

(1) $$2\mathrm{ZnS}+3O_{2}=2\mathrm{ZnO}+2{\mathrm{SO}}_{2}\;\Delta rG_{m,1}$$

(2) $$C+O_{2}={\mathrm{CO}}_{2}\;\Delta rG_{m,2}$$

The change of Gibbs free energy can be used as a criterion for the spontaneous process of thermochemical reactions in a closed system with isothermal pressure and without non-volume work. When the change of Gibbs free energy for the reaction is negative, the reaction proceeds spontaneously, and when the change of Gibbs free energy for the reaction is zero, the reaction reaches equilibrium. While the change of Gibbs free energy for the reaction is positive, the reaction cannot proceed. As shown in Table S2, the value of $\Delta rG_{m,1}$ and $\Delta rG_{m,2}$ are calculated to be negative, implying that the reaction thermodynamics of oxidations of zinc sulfide and carbon can be carried out. Combined with the results of EA, we consider that the formation of the core shell structure is related to the competition between these two reactions, and the selectivity may be closely related to their reaction rates.

**Figure 4:**
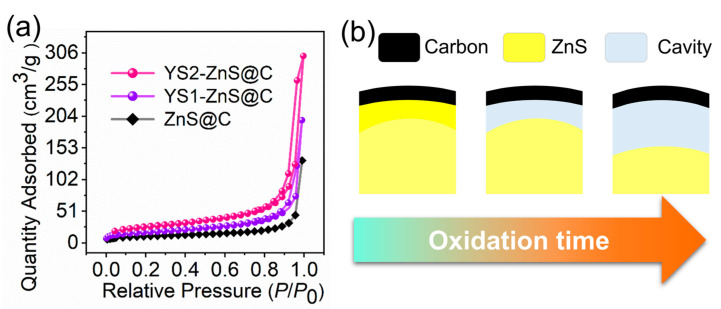
(**a**) Nitrogen adsorption-desorption isotherms of ZnS@C, YS1-ZnS@C and YS2-ZnS@C. (**b**) Schematic of controlling size of cavity of materials by oxidation time.

From the results of nitrogen adsorption-desorption isotherms shown in Figure 4a and Table S3, the samples of YS1-ZnS@C and YS2-ZnS@C show a typical type IV isotherm with H3 hysteresis loop based on the IUPAC’s classification of the characteristic curves of mesoporous materials, while the sample of ZnS@C exhibits a type II isotherm without adsorption-desorption hysteresis associated with the characteristics of non-porous materials [42,43]. The characteristic of H3 hysteresis loop implies the formation of slit-shaped pores in the YS-ZnS@C after the removal of surface ZnO [43,44]. The Brunauer–Emmett–Teller (BET) surface area of the YS1-ZnS@C and YS2-ZnS@C are measured to be 60.4 and 92.1 m^2^ g^−1^, respectively, which are much higher than that of the ZnS@C (36.5 m^2^ g^−1^). The larger specific surface area of YS-ZnS@C is essential for enhancing the power/energy density of LIBs [44]. Meanwhile, the pore volume increased from 0.205 cm^3^ g^−1^ for ZnS@C to 0.305 cm^3^ g^−1^ for YS1-ZnS@C and 0.465 cm^3^ g^−1^ for YS2-ZnS@C with the increase of oxidation duration, confirming that the size of the cavity can be controlled by changing the content of ZnO via regulating the oxidation duration (Figure 4b). The high specific surface area equipped with the large pore volume of YS-ZnS@C is benefit to the formation of a solid electrolyte interface (SEI) film and the infiltration of electrolyte, thus alleviating the volume expansion of lithium ion embedding [45]. As a result, excellent performances of YS-ZnS@C for LIBs can be expected.

**Figure 5:**
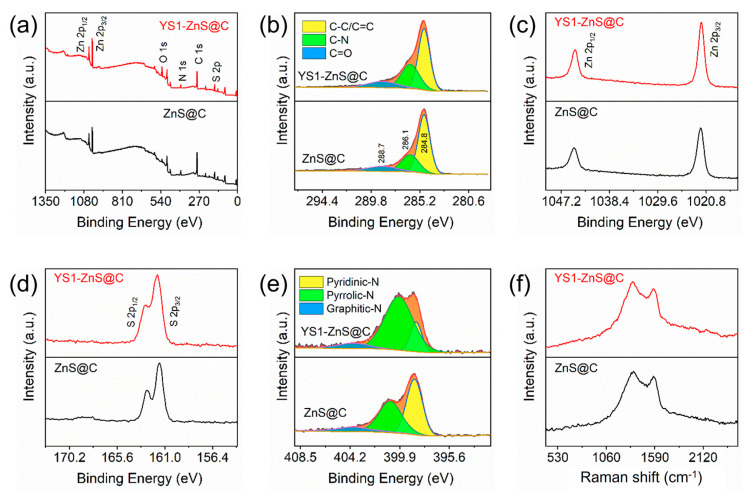
XPS spectra of (**a**) survey, (**b**) C 1s, (**c**) Zn 2p, (**d**) S 2p, and (**e**) N 1s. (**f**) Raman spectra for ZnS@C and YS1-ZnS@C composite.

The composition and chemical bonding states of ZnS@C and YS1-ZnS@C have been performed by the XPS spectroscopy. Zn, S, C, N, and O elements can be easily observed in the survey XPS spectra of ZnS@C and YS1-ZnS@C (Figure 5a), corresponding to their chemical composition; the small amount of O element possibly originates from adsorbed oxygen. From Figure 5b, the C1s spectra were fitted into three peaks located at 284.8, 286.1, and 288.7 eV, which are identified as C-C/C=C, C-N, and C=O, respectively [46,47]. As shown in Figure 5c, it can be found that the high-resolution Zn 2p XPS spectra were divided into two peaks located at 1045.3 and 1022.3 eV, which are attributed to Zn 2p_1/2_ and Zn 2p_3/2_ states, respectively. The high-resolution S 2p XPS spectra in the Figure 5d reveal two peaks centering at 163.4 and 162.2 eV, which are ascribed to S 2p_1/2_ and S 2p_3/2_, respectively. The fitted peaks centering at 398.5, 400.0, and 403.9 eV in the N 1s XPS spectra (Figure 5e) are assigned to pyridine-N, pyrrole-N, and quaternary-N, respectively [48]. Moreover, Raman spectroscopy is further used for investigation. As shown in Figure 5f, the graphitic crystallite-derived G band and the defect-induced D band in the ZnS@C and YS1-ZnS@C have been detected at around 1533 and 1351 cm^−1^, respectively [49,50]. The calculated intensity ratios of I_D_/I_G_ for ZnS@C (1.05) and YS1-ZnS@C (1.04) remain high and almost equal, further demonstrating that the nitrogen-doped carbon polyhedral in the YS1-ZnS@C keeps the amorphous feature, which is conducive to enhancing conductivity [51].

**Figure 6:**
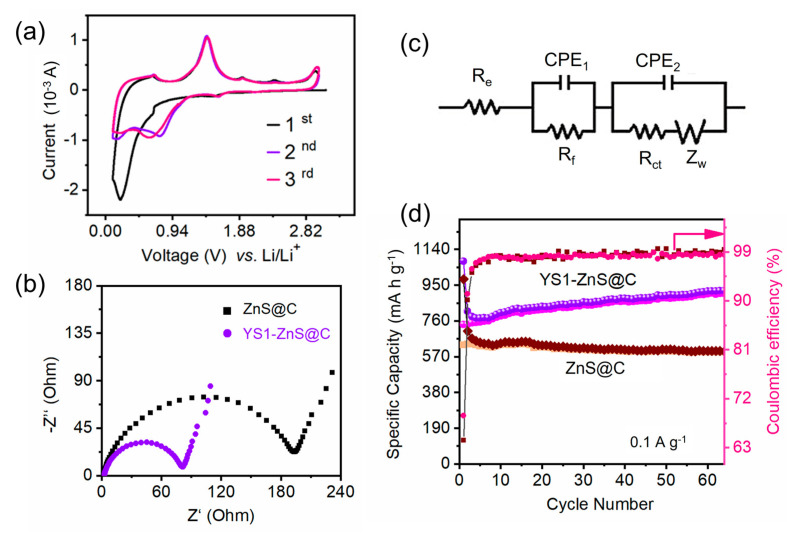
(**a**) Cyclic voltammogram curves of YS1-ZnS@C in the initial three cycles at 0.2 mV s^−1^ between 0.1 and 3.0 V. (**b**) Nyquist plots and (**c**) the equivalent circuit model [54] of ZnS@C and YS1-ZnS@C electrodes; (**d**) Cycling performance of ZnS@C and YS1-ZnS@C electrodes in the voltage range of 0.1–3.0 V at a current density of 100 mA g^−1^.

The lithium storage performances were tested by CR 2032 half cells. Figure 6a and Figure S3 show the profiles of ZnS@C and YS1-ZnS@C at the first three cycles with a scan rate of 0.2 mV s^−1^ at the voltage of 0.1–3.0 V vs. Li/Li^+^. Both ZnS@C and YS1-ZnS@C show good cycling stability after the first cycle due to the contribution of a strong conductive carbon layer [40]. In the first cathodic scan, the broad peaks at ca. 0.25 V are ascribed to the multiple reactions (ZnS + 2 Li^+^ + 2 e^−^→ Zn + Li_2_S, Zn + *x* Li^+^ + *x* e^−^ → Li*_x_*Zn) and the production of SEI [52], which would disappear in the following cycles. The peaks centering at ca. 0.67 and 1.41 V during the following scan are related to the multistep dealloying reaction of Li-Zn alloy (Li*_x_*Zn → *x* Li^+^ + Zn + *x* e^−^) and the regeneration of ZnS (Zn + Li_2_S → ZnS + 2Li^+^ + 2 e^−^), respectively [22,53].

Figure 6b shows the Nyquist plots of ZnS@C and YS1-ZnS@C electrodes. The equivalent circuit model is presented in Figure 6c on the basis of previous reports [54,55,56]. *R*_e_ represents the internal resistance; *R*_f_ and CPE_1_ corresponding to high frequency semicircles are related to the resistance and constant phase element of SEI film, respectively; *R*_ct_ and CPE_2_ related to high-medium frequency semicircles correspond to the resistance of charge-transfer and constant phase element of interface between electrode and electrolyte, respectively; Z_W_ is related to the Warburg impedance, corresponding to the diffusion process of lithium ion in low frequency. Modeling AC impendence spectra based on the equivalent circuit were further used to explore the kinetic differences of electrodes. The fitted results indicate that the charge-transfer resistance (*R*_ct_) of YS1-ZnS@C (64 Ω) and ZnS@C (62 Ω) remain at a low level. Furthermore, the SEI film resistance (*R*_f_) of YS1-ZnS@C is 6.3 Ω, which is significantly smaller than that of ZnS@C (123.0 Ω). This fact confirms that the high specific surface area equipped with the large pore volume brought by this manufacturing gap strategy can form a low resistance SEI film to improve conductivity and accelerate electron transport during the electrochemical reversible reactions.

**Figure 7:**
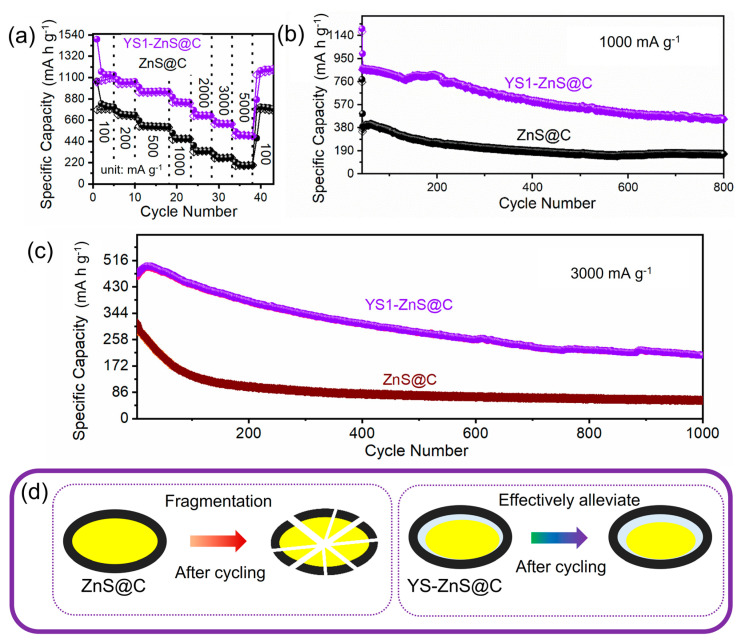
(**a**) Rate performances and long-term cycling performances at (**b**)1000 and (**c**) 3000 mA g^−1^ for YS1-ZnS@C and ZnS@C. (**d**) Schematic illustrations of structure changes after cycles.

Figure 6d shows the cycling performances of the ZnS@C and YS1-ZnS@C electrodes at a current density of 100 mA g^−1^. As shown in Figure S4, due to the formation of irreversible SEI for the YS1-ZnS@C electrode, YS1-ZnS@C delivers a high initial discharge capacity of 1076 mA h g^−1^ and a charge capacity of 741 mA h g^−1^, which corresponds to a low coulombic efficiency (CE) of 68.85% (64.34% for ZnS@C) [57]. At the second and third cycles, the capacity loss mainly originates from the formation and dissolution of intermediate metal sulfides in the electrolyte. As the number of cycles increases, the stable SEI film has been obtained, and the formation and dissolution of intermediate metal sulfides in the electrolyte reach equilibrium gradually, which results in the increasing of the Coulombic efficiency of YS1-ZnS@C electrodes, nearly 100% after ten cycles. Furthermore, the specific capacity of YS1-ZnS@C electrodes is obviously higher than that of ZnS@C, which is mainly attributed to the more reactive active sites brought about by the smaller size and larger surface area of YS1-ZnS@C. The cycling stability of ZnS@C is poor, and the discharge capacity only maintains 604 mA h g^−1^ after 65 cycles (~61% of the initial discharge capacity). Interestingly, the excellent cyclic stability can be observed in the YS1-ZnS@C. The reversible capacity of the YS1-ZnS@C electrode does not decrease but slightly increase. After 65 cycles, the discharge capacities of 910 mA h g^−1^ have remained in the YS1-ZnS@C electrodes (about 2.5 times the theoretical capacity (372 mA h g^−1^) of commercial graphite anodes), with retention of 84%. This interesting phenomenon may be caused by the recovery process after the initial SEI formation. The excellent cycling behavior of the YS1-ZnS@C is also confirmed in the rate performances as shown in Figure 7a. It depicts that the reversible capacities over YS1-ZnS@C are ca. 1127, 1058, 959, 843, 709, and 621 mA h g^−1^ at a current density of 100, 200, 500, 1000, 2000, and 3000 mA g^−1^, respectively. While performing at the extremely high current density of 5000 mA g^−1^, YS1-ZnS@C keeps a high capacity of 499 mA h g^−1^ while ZnS@C only maintains a reversible capacity of 195 mA h g^−1^. When changing back to 100 mA g^−1^, the capacity of YS1-ZnS@C surprisingly recovers to 1195 mA h g^−1^, which is much higher than that of ZnS@C (777 mA h g^−1^). It mainly benefits from the significant alleviation of volume expansion for YS1-ZnS@C during Li^+^ embedding due to the presence of the cavities. After the rate performance testing, the electrodes are further examined regarding long-term cycle performance at 1000 mA g^−1^ (Figure 7b). When the cycles are at 200, the YS1-ZnS@C could hold a capacity of 811 mA h g^−1^, while the capacity of ZnS@C decays to 256 mA h g^−1^. Even after 800 cycles, the YS1-ZnS@C still maintains a reversible capacity of 450 mA h g^−1^ (it has decayed to 163 mA h g^−1^ for ZnS@C) with a CE of nearly 100%.

Moreover, the long-term cycling stability of electrodes have been also performed at a high current density of 3000 mA g^−1^ (Figure 7c). In order to better exert the electrochemical performance at high current density, the electrodes were activated at a low current density of 100 mA g^−1^ to form a stable SEI film (Figure S4). The initial charge capacity of YS1-ZnS@C electrodes at 3000 A g^−1^ is 459 mA h g^−1^, and its capacity after 20 cycles slightly increases to 496 mA h g^−1^ (Figure 7c). In addition, long-term cycling performance at 3000 A g^−1^ reveals the excellent stable cycling performance of YS1-ZnS@C, and a high reserved capacity of 206 mA h g^−1^ is still delivered after 1000 cycles. Such high-capacity retention manifests the outstanding cycling stability of YS1-ZnS@C, which can stem from its new and unique structure design. By contrast, the capacity of ZnS@C is severely degraded, leaving only 60 mA h g^−1^ after 1000 cycles (ca. 1/3 of capacity of YS1-ZnS@C). One of the possible reasons is the different structural adjustments of ZnS@C and YS1-ZnS@C in response to inevitable volume expansion (Figure 7d). For the ZnS@C electrode, although it is initially wrapped with a highly conductive carbon layer, the carbon layers are unable to sustain the huge distortion of ZnS during rapid repeated reversible reactions and undergo fragmentation due to the lack of the internal gap, leading to the low conductivity of ZnS [24]. Comparatively, yolk-shell structure of YS1-ZnS@C contains the internal clearances that can effectively alleviate volume expansion, release enormous stress, and provide a great deal of active sites, exhibiting outstanding mechanical strength as well as alleviating the collapse and aggregation of ZnS during fast charge-discharge process.

## 4. Conclusions

In summary, carbon-coated ZnS yolk-shell structures have been prepared by the temperature-controlled partial selective oxidation of solid core-shell structured ZnS@C precursor and subsequent acid etching. When acting as an anode material for LIBs, the as-prepared YS1-ZnS@C composite exhibits an exceptional discharge specific capacity of 910 mA h g^−1^ at the current density of 100 mA g^−1^, while ZnS@C only exhibits 604 mA h g^−1^. Notably, at a large current density of 3000 mA g^−1^, a capacity of 206 mA h g^−1^ can still be maintained for YS1-ZnS@C a composite after 1000 cycles, which is over three times the capacity of ZnS@C. The large difference in lithium storage performance for ZnS@C and YS1-ZnS@C is due to the former having low surface area and substantial volume variation, while the latter possesses the cavity and large surface area that provides abundant active sites and effectively alleviate the volume expansion issue in the charge-discharge process. This work provides a new way to solve the volume expansion and low conductivity of metal chalcogenides anode materials in LIBs. It is expected that more metal chalcogenides with high specific capacity and excellent cycling stability can be obtained via this synthetic strategy.

## Data Availability

All data used in this work will be available upon reasonable request.

## Supplementary Materials

The following supporting information can be downloaded at: https://www.mdpi.com/article/10.3390/ma16052097/s1. The details of ZnS@C material synthesis. Characterization and electrochemical measurements parameters. Figure S1: TEM images of (a) ZnS@C, (b) ZnS/ZnO@C, and (c) YS1-ZnS@C. HRTEM images of (d) ZnS@C, (e) ZnS/ZnO@C-1, and (f) YS1-ZnS@C. Figure S2: (a)XRD patterns of ZnS@C, ZnS/ZnO@C-2, and YS2-ZnS@C. (b) XRD patterns and photos for the pristine ZnS@C sample and the ZnS@C samples oxidized at different temperatures; Figure S3: Cyclic voltammogram curves of ZnS@C at the initial three cycles with a scan rate of 0.2 mV s^−1^ between 0.1 and 3.0 V vs. Li/Li^+^; Figure S4: The activations of ZnS@C and YS1-ZnS@C at the low current density of 0.1 A g^−1^; Table S1: The EA results of samples; Table S2: The thermodynamic data. Table S3: The nitrogen adsorption-desorption isotherms results.
