# Supplementary material for: A Selective Oxidation Strategy towards the Yolk–Shell Structured ZnS@C Material for Ultra-Stable Li-Ion Storage
Source: Materials (Basel). 2023 Mar 4;16(5):2097. doi: 10.3390/ma16052097 (PMC10004707; doi:10.3390/ma16052097)
Supplement: Supplementary file 1 [file materials-16-02097-s001.zip › materials-2202619-supplementary.pdf]

Supporting information

A selective oxidation strategy towards the yolk–shell structured ZnS@C material for ultra-stable Li-ion storage

Wenhua Liao[1,2], Qianqian Hu[1,*], Xiaoshan Lin[1], Ruibo Yan[1,2], Guanghao Zhan[1], Xiaohui Wu[2] and Xiaoying Huang[1,*]

Materials

Zinc sulfide ($ZnCl_2$, AR), Acetic acid (HAc, AR) and Ethanol (CH_3CH_2OH, 95%) were purchased from Sinopharm Chemical Reagent Co., Ltd. (Shanghai, China). Sulfur (S, 99.9%) was purchased from Tianjin Komio Chemical Reagent Co., Ltd. (Tianjin, China). n-butylamine (ba, 99.0%) was purchased from Shanghai Adamas Reagent Co., Ltd. (Shanghai, China). All the chemicals were used without further purification. Deionized water was obtained by reverse osmosis by filtration and ion exchange (YA. ZD-5 controlled type, Shanghai Shen An Medical Instrument Factory).

Synthesis of ZnS@C material with core shell structure.

In a typical synthesis, 0.64 g (20 mmol) of S, 5.46 g (40 mmol) of $ZnCl_2$ and 70 mL of *n*-butylamine (ba) were added into a 100 mL of round bottom Flask. After heating in an oil bath at 65 ^{o}C for 4 h in N_2 atmosphere with a magnetic stirring of 500 rpm, the mixture was cooled down to room temperature (RT) naturally with a continuous stirring. The obtained white powder was washed several times with water and ethanol and collected by centrifugation. After drying the precipitate at 55 ^{o}C in vacuum, the obtained while powder (20 – 50 mg) was encapsulated in a quartz tube with diameter of 10 mm and length of ca. 15 cm which was then evacuated and sealed. The tube was heated to 700 ^{o}C with heating rate of 2 ^{o}C min^{-1}, which was then kept at 700 ^{o}C for 2 h in the muffle furnace. Finally, the black powder of ZnS@C material with core shell structure was obtained.

Characterization.

X-ray diffraction (XRD) patterns were performed on a Rigaku X-ray MiniFlex II diffractometer with Cu *Ka* radiation in the range of 10 to 60 °. The morphology was investigated by Field Emission Scanning Electron Microscope (FESEM) on a SU-8010 operating at 5 kV, and Transmission Electron Microscope (TEM) and High-Resolution Transmission Electron Microscope (HRTEM) on a FEI Tecnai F20 operating at 200 kV. Raman spectra were measured by using a FTIR-Raman spectrometer (Horiba Labram HR800 Evolution) with a wavelength of 532 nm. X-ray photoelectron spectroscopy (XPS) was conducted on a Thermo Fisher ESCALAB 250Xi by using an X-ray source (Al K_{α}, λ = 8 Å, $h\nu$ = 1486.6 eV) without any etching. Elemental analysis (EA) was measured by Elementar Vario Elcube. Nitrogen adsorption/desorption isotherms were measured with an ASAP 2020 based on the Brunauer-Emmett-Teller (BET) method.

Electrochemical measurements.

Electrochemical measurements were performed with CR2032 coin-type cells. The working electrodes consisted of the active material (80 wt%), carbon black (10 wt%) and the carboxymethyl cellulose (CMC) (10 wt%). The mass loading on the surface of the copper foil with a diameter of 12 mm was ca. 1.1 mg cm^{-2}. Lithium foil was used as the counter and reference electrode, and Celgard 2325 membrane was used as the separator. The electrolyte was 1 M $LiPF_6$ in ethylene carbonate/dimethyl carbonate/diethyl carbonate (1 : 1 : 1 in volume) with a 5 vol% fluoroethylene carbonate. Electrochemical measurements were performed on a LAND 2001A test system with the voltage range of 0.1 – 3.0 V at 30 ^oC. The electrochemical impedance spectroscopy (EIS) with an alternating current amplitude of 5 mV, frequency ranging from 1 Hz to 100 kHz and cyclic voltammogram (CV) in the voltage range of 0.1 – 3.0 V was performed on a CHI660E electrochemical workstation.

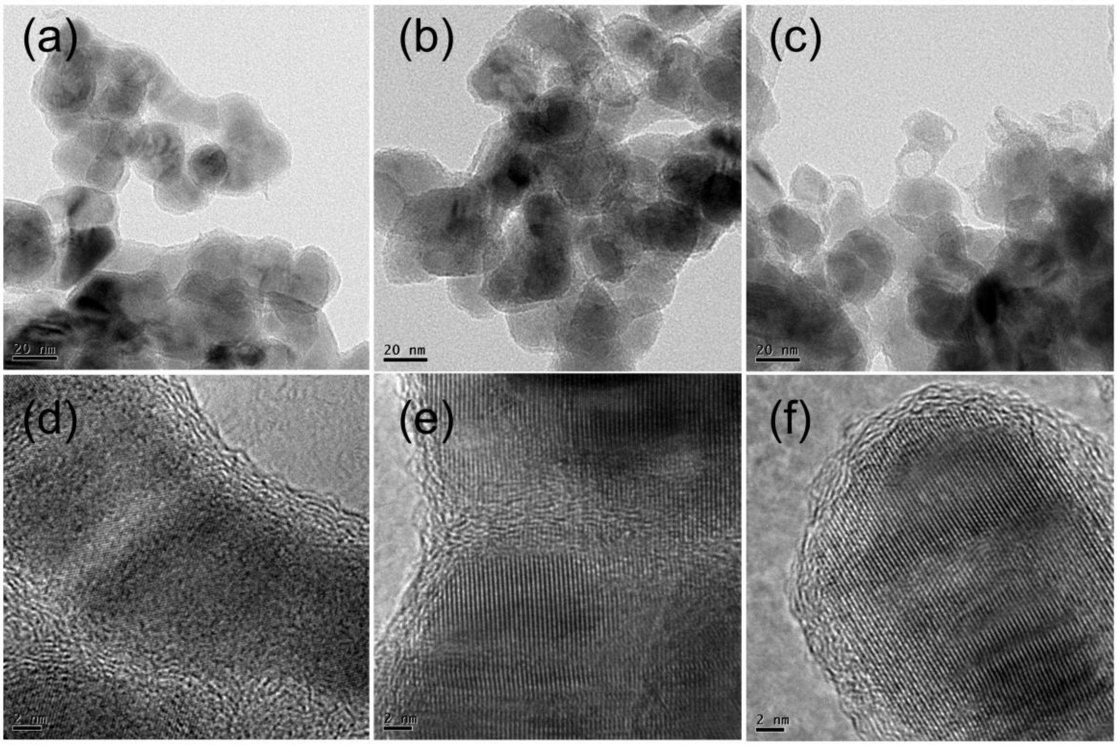

Figure S1. TEM images of (a) ZnS@C, (b) ZnS/ZnO@C-1, and (c) YS1-ZnS@C. HRTEM images of (d) ZnS@C, (e) ZnS/ZnO@C-1, and (f) YS1-ZnS@C.

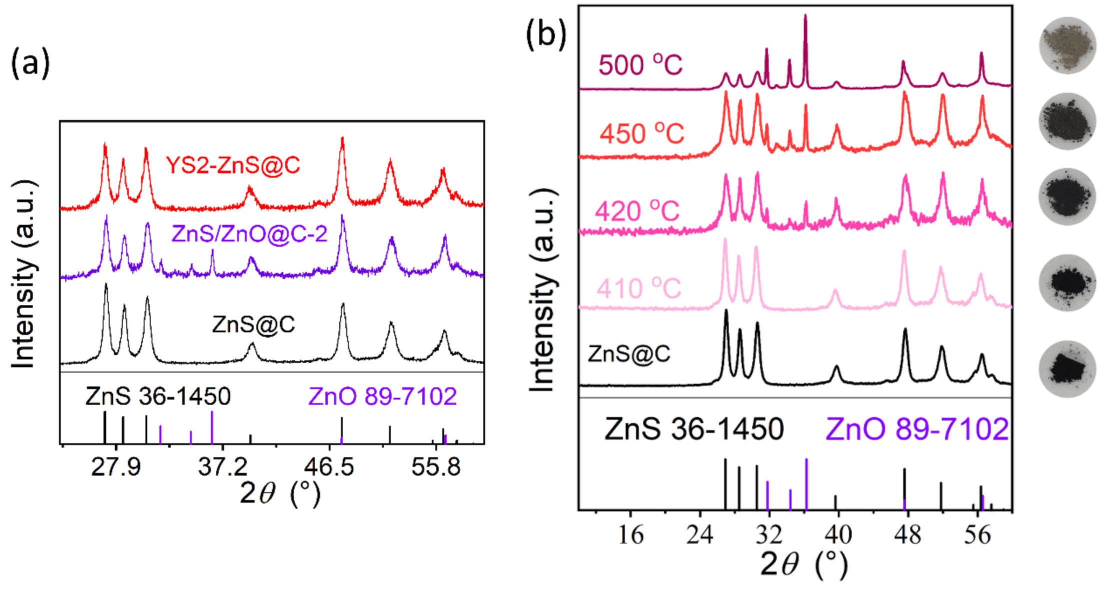

Figure S2. (a)XRD patterns of ZnS@C, ZnS/ZnO@C-2, and YS2-ZnS@C. (b) XRD patterns and photos for the pristine ZnS@C sample and the ZnS@C samples oxidized at different temperatures.

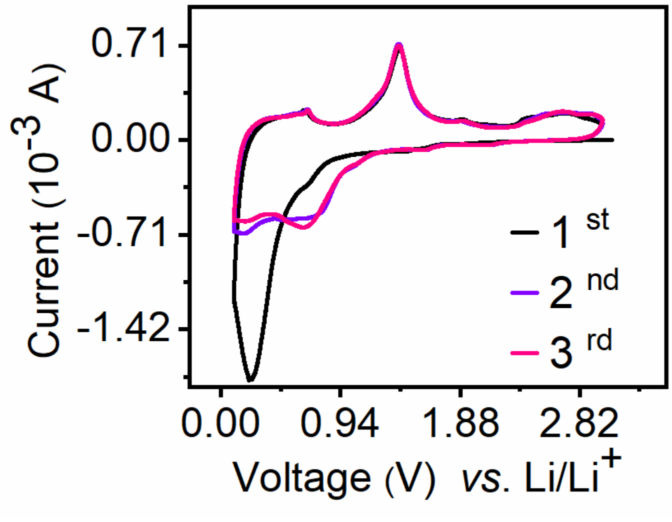

Figure S3. Cyclic voltammogram curves of the ZnS@C material at the initial three cycles with a scan rate of 0.2 mV s^{-1} between 0.1 and 3.0 V *vs.* Li/Li^+.

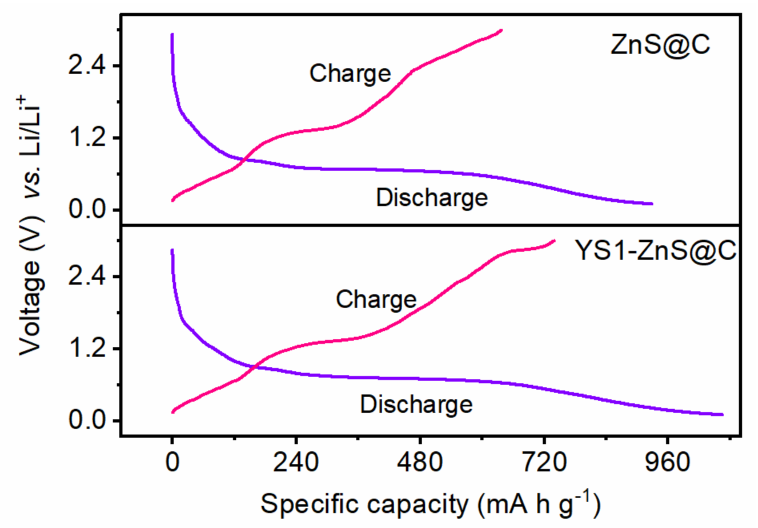

Figure S4. The activations of ZnS@C and YS1-ZnS@C at the low current density of 0.1 A g^{-1}.

Table S1. The EA results of samples.

Sample	S wt%	O wt%	C wt%	N wt%
**ZnS@NC**	26.6	2.0	12.0	2.7
**ZnS/ZnO@C-1**	26.6	5.8	10.1	2.1
**ZnS/ZnO@C-2**	27.0	6.6	7.8	1.8

Table S2. The thermodynamic data.

Substance	State	$\Delta_f G_m^\ominus$ (kJ mol^{-1})
**ZnS (Würtzite)**	Solid	-242.5
**O_2**	Gaseousness	0
**ZnO**	Solid	-318.3
**SO_2**	Gaseousness	-300.2
**C**	Solid	0
**CO_2**	Gaseousness	-394.4
	**Calculated value (kJ mol^{-1})**	
$\Delta rG_{m,1}$	-752	
$\Delta rG_{m,2}$	-394.4	

Table S3. The nitrogen adsorption-desorption isotherms results.

**Sample**	**Isotherm type**	**Hysteresis loop**	**Specific surface area (m^2 g^{-1})**	**Pore volume cm^3 g^{-1}**
**ZnS@C**	II	/	36.5	0.205
**YS1-ZnS@C**	IV	H3	60.4	0.305
**YS2-ZnS@C**	IV	H3	92.1	0.465
